# Can extubation failure be related to high unit activity?

**DOI:** 10.1186/cc9587

**Published:** 2011-03-11

**Authors:** I Keith, R Sundaram, KD Rooney

**Affiliations:** 1 RAH, Glasgow, UK

## Introduction

Extubation failure has become an important quality indicator. The aim of our study was to ascertain whether extubation failure was related to unit activity; that is, whether it was more frequent on days of greater unit activity.

## Methods

We retrospectively analysed 520 consecutive admissions to our seven-bed ICU over an 18-month period. We defined extubation failure as the need for reintubation within 24 hours. Bed occupancy was used as a surrogate marker of unit activity. Bed occupancy was based upon the number of hours patients were nursed in the ICU each day and was summed and expressed as a percentage of the maximum available (24 × 7). Data were collected from our national audit database and analysed using SPSS software.

## Results

**Figure 1:**
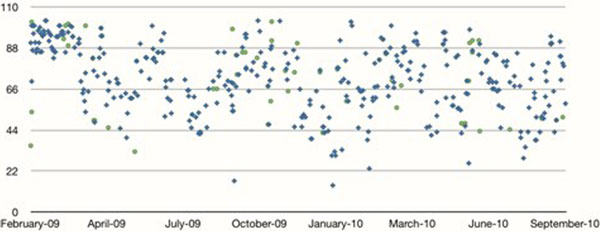
**Scatterplot of reintubation rates versus bed occupancy**.

We studied 520 intubated patients over an 18-month period after excluding children, tracheostomised patients and patients who were receiving end-of-life care. Sixty-five patients (12.5%) were reintubated within the 24 hours. Bed occupancy was not different in the extubation success group as compared with the failure group (70.6 CI ± 1.75 vs. 72.9 CI ± 4.9; *P *= 0.37). The two groups were similar in terms of their severity of illness; that is, APACHE II scores. Length of stay was increased in the extubation failure group. There was no correlation between bed occupancy and extubation failure using the Pearson correlation coefficient (*R *= 0.05; *P *= 0.68). See Table 1 and Figure 1.

**Table 1:** 

	Control	Failure	*P *value
Number	455	65	
Age	57.26	51.2	0.01
APACHE	20.33	21.5	0.69
Bed occupancy	70.6	72.9	0.37
Admitted	1.21	1.21	0.84
Discharged	1.4	1.32	0.37

## Conclusions

We could not demonstrate any correlation between high unit activity and reintubation rates.
